# Supplementary material for: Are carbon emissions trading and green financial instruments synergistic? -Comprehensive quantitative research based on content analysis
Source: PLoS One. 2024 Mar 7;19(3):e0298601. doi: 10.1371/journal.pone.0298601 (PMC10919601; doi:10.1371/journal.pone.0298601)
Supplement: S1 Data — (ZIP) [file pone.0298601.s001.zip › Data/地级市Z_F工作报告.zip!/╣≤╓▌/╣≤╤⌠╩╨/└┤╫╘╣1⁄2╓┌║┼í╛┬φ┐╦╩2╛▌═°í┐_2004.docx]

各位代表：

现在，我代表市人民政府向大会作工作报告，请各位代表予以审议，并请各位政协委员和其他列席人员提出意见。

一、2003年工作回顾

2003年是本届政府任期的第一年。一年来，我们在省委、省政府和市委的正确领导下，以“三个代表”重要思想和十六届三中全会精神为指导，解放思想，抢抓机遇，开拓创新，扎实工作，努力克服“非典”疫情和各种困难带来的影响，圆满完成了市十一届人大二次会议确定的各项目标任务，全市经济保持了持续快速健康协调发展的良好势头，呈现出增长比较快、效益比较好、活力比较强的特点，各项社会事业都取得了新的成绩。

（一）国民经济持续快速增长，综合经济实力显著增强

2003年全市生产总值预计完成380.92亿元，比上年增长13%，分别高于全国、全省平均水平3.9和2.9个百分点。其中，第一产业增加值28.81亿元，增长6.7%；第二产业增加值191.94亿元，增长14.8%；第三产业增加值160.17亿元，增长11.8%；三次产业结构比重由上年的7.9：50.9：41.2调整为7.6：50.4：42，产业结构日趋合理。财政总收入完成91.6亿元，增长15.8%，其中地方财政收入完成40.39亿元，增长22.1%；固定资产投资完成239.9亿元，增长27.6%，净增51.94亿元。城市居民人均可支配收入达7985元，增长9.3%，农民人均纯收入达2507元，增长6.6%。全市金融机构各项存款余额达893.6亿元，增加161.81亿元，贷款余额达763.61亿元，增加168.07亿元。区县经济发展势头强劲，我市列入第一轮强县建设的7个区、市、县中，云岩、南明、白云、乌当综合排位名列全省前四名。我市经济结构调整的成效正逐步显现出来，经济进入快速健康发展时期。

（二）经济结构调整扎实推进，经济整体素质全面提升

农业结构调整力度加大，农村经济全面发展。以农业产业化经营为主线，以农产品质量标准体系建设为突破口，大力发展效益型农业，着力依托市场、立足资源、围绕特色调整农村经济结构，在稳定粮食生产的基础上，大力发展畜牧业、药业、花卉产业，提高农产品商品率，有力地促进了第一产业稳步发展。种植业结构调整进一步向市场化、区域化和优质、特色转变，继续呈现粮食播种面积稳定，经济作物、特色产品、无公害农产品播种面积增加的态势，全市粮食总产量达56.35万吨，增长28.9%，油菜籽产量达5.46万吨。畜牧业得到大力发展，产值预计占农业总产值比重达38.5%。狠抓优质烤烟基地建设，烤烟生产创1997年以来最好水平，产量达1.66万吨，增长8.9%。农村基础设施建设进一步加强，提高了农业农村抵御自然灾害的能力，完成花溪水库扩建加高、小关水库改造工程；完成人饮工程199处，解决7万农村人饮困难，建成56处无公害农产品、中药材基地配套水利设施；完成沼气池、改圈、改厕各2万个，坡改梯1.61万亩，茅草房改造8183户。通过“四改一气”及水、电、路、房等配套设施的建设和改造，改善了农村生产生活条件。乡镇企业在结构调整中保持稳定增长，预计实现乡镇企业增加值120.61亿元，增长18.59%，实交税金8.45亿元，增长28.6%。

工业经济继续保持稳定增长，经济运行的质量和效益大幅提高。预计全市工业增加值完成151.62亿元，增长14.1%，市属扭亏增盈考核企业盈亏相抵后实现利润3238万元。工业产品产销衔接良好，产销率达96.7%，工业经济综合效益指数为111.4%，利润总额增长158.6%。全市工业对经济增长的贡献率达45.3%，拉动经济增长5.9个百分点。市属国有及国有控股工业企业连续三年实观盈利。企业信用担保服务体系建设步伐加快。鼓励企业技术创新、技术改造，技改投入力度加大，全年共完成重点技改项目20项，完成更新改造投资53.45亿元。贵州水晶有机化工集团有限公司聚乙烯醇2改3万吨／年技术改造项目、贵阳海信电子有限公司海信工业园老线搬迁及新线建设项目、南方汇通股份有限公司的1英寸微硬盘等项目均已实现投产。通过加大工业结构调整和技术改造力度，企业竞争力明显增强。

第三产业日益活跃，整体水平得到提升。金融保险、房地产业、教育文化、交通运输业、餐饮业、娱乐业等传统产业继续稳步发展，规模不断扩大。物流配送、通信、网络、信息咨询和社区服务业等新型产业发展迅速。消费品市场繁荣，商品供应丰富，物价平稳，全市社会消费品零售总额完成153.67亿元，比上年增长12.5%，居民消费价格指数为100.8%。旅游基础设施投入和对外宣传力度加大，旅游业呈现良好的发展势头，预计全年共接待国内游客921.59万人（次），海外游客2.26万人（次），旅游收入达38.74亿元，第三产业对国民经济的贡献作用进一步增强。

非公有制经济快速发展，效益不断提高。制定了《关于加快发展非公有制经济的若干措施》和《贵阳市促进非公有制经济发展表彰奖励办法》，建立了非公有制经济发展投诉中心，扩大了非公有制企业贷款担保面，为非公有制经济的发展营造良好环境，促进了非公有制经济的快速发展。老干妈、西洋肥业、益佰制药、益康制药等一批非公有制企业利税大幅上升，为我市经济发展做出了积极的贡献。预计全市非公有制经济实现增加值147.85亿元，增长22%，占生产总值的比重达38.8%。

（三）各项改革稳步推进，对外开放向纵深发展

以国有企业改革为重点，深化各项改革。坚持把加快改革作为促进经济发展的根本动力，出台了《关于大力推进国有资产重组和国有企业改革的实施意见》，大力推进以产权制度为重点的企业改革，国有企业在体制创新、机制转换和结构优化上取得重大进展。贵州玻璃厂、贵州朝晖机械厂依法破产，中国七砂、贵阳电线厂、服务大楼资产重组基本完成。积极引导省内外有实力的企业和经营者参与我市企业改革改制，成功实现了小河污水处理厂资产转让、市煤气公司改制、金筑酒店股权转让，人民广场地下商场成功引进世界500强企业沃尔玛公司入驻经营，市政公用事业改革实现了“零”的突破。农村税费改革逐步规范完善，农民负担进一步减轻，全年共减负8006.67万元，减负率达60.56%。国有粮食购销企业改革稳步推进，减员分流工作全面完成。农村信用社改革试点工作进展顺利。社会保障体系框架基本建立。事业单位人事制度改革扎实推进，完成了30个事业单位的人事制度改革试点工作。行政审批制度改革继续深入，共取消行政审批项目117项。投融资体制改革迈出新步伐。经过艰苦努力，我市与国家开发银行达成协议，争取到国家开发银行在5年内向我市提供150亿元的授信贷款，为我市今后几年的建设和发展提供了重要资金保障和支撑。

对外开放进一步扩大，招商引资工作取得新突破。坚持“环境立市”战略，招商引资意识增强，投资环境明显改善。出台了《贵阳市招商引资目标责任考核及奖惩试行办法》、《关于进一步加大招商引资工作力度的若干规定》等一系列促进招商引资的政策和措施，明确目标，创新方法，建章立制，招商引资力度进一步加大，安商、稳商、富商、以商招商工作得到加强，招商引资呈现出前所未有的良好势头。引进了中化集团、娃哈哈、青岛汉缆等一批企业集团在我市投资兴建项目，为经济发展增强了后劲。全年引进内资实际到位资金90.26亿元，增长1.3倍；直接利用外资4450万美元，增长52.5%；外贸进出口总额完成8.52亿美元，增长42.2％，创历史最好水平。

（四）基础设施建设步伐加快，城市管理水平不断提高

城市建设力度进一步加大。坚持推进新区建设、旧城改造、小城镇建设“三轮驱动”战略，城市化水平不断提高。编制完成市域城镇体系规划、近期建设规划和32个乡（镇）的控制性详细规划。全年基本建设投资完成104.45亿元，实施重点基础设施工程项目39个。金阳新区在完成道路主骨架建设的基础上，将工作重心转移到人口聚集和产业支撑上来，开工建设了新区住宅、医院、学校等配套项目，连接市区和金阳新区的小关特大桥顺利贯通，建成市级行政中心，市四大班子顺利实现搬迁。老城区改造以完善和提升城市功能为核心，抓好市区道路改造、停车场、污水处理厂等一批重点基础设施建设。完成了文化路、安云路、青云路、护国路等10条城市道路建设，西出口线、中心环北线、艺校铁路立交桥、花溪大道南段的建成使用，不仅有效地缓解了城市道路“瓶颈”问题，而且使城市的骨架进一步完善、特色更加鲜明。启动“城中村”改造工作，清理烂尾楼和闲置用地工作取得重大进展。小城镇建设全面加速，区、市、县政府所在地和一批中心集镇的基础设施不断完善。超额完成500公里县乡道路改造任务，城乡交通状况明显改善。

城市管理工作进一步加强。开展了以“争做文明市民，创建优美环境”为重点的城市市容市貌以及“畅通工程”等综合整治活动，市容市貌和交通秩序得到明显改观，使城市管理水平迈上了新台阶。严格实行经营性土地公开招标拍卖，全年共出让土地109宗，查处违法用地376宗。城市生态绿化建设进展顺利，完成二环林带造林7.77万亩，天保人工造林5万亩，新增城市绿地面积102.8万平方米。“南明河三年变清”系列工程强力推进，今年汛期前后可基本实现“水变清、岸变绿、景变美”的目标。提请市人大常委会制定实施了《南明河保护管理办法》。通过“南明河三年变清”环境综合整治工程的实施，改善了城市环境质量，城市形象和品位进一步提升。

（五）各项社会事业协调发展，精神文明建设和民主法制建设全面推进

社会各项事业全面进步。坚持“科教兴市”战略，组织实施科技项目120项，成果鉴定32项，推广了一批关键技术和先进实用技术，高新技术产业园、科技示范园、知识经济产业园区发展步伐加快。积极推进电子政务建设，企业信息化“1050”工程取得实质性进展，全面启动“数字金阳”和贵阳地理信息系统一期工程，开通了贵阳市远程教育网络。继续巩固和提高“两基”达标成果，稳步推进基础教育课程改革、中小学布局调整，全面普及九年制义务教育，启动了“退高进初”工作，高中招生规模进一步扩大，完成了高中扩招20%的任务。文化产业稳步发展，城市文化品位进一步提升，先进文化对经济社会的影响力日益增强，成功举办了“林城读书月”、“广场文艺演出”等群众性文化活动。加大了对县乡卫生基础设施的投入力度，建立健全了市、县、乡、村四级疾病预防控制体系。人口与计生工作继续加强，人口自然增长率控制在8.2‰以内。资源保护和开发利用扎实推进。与此同时，统计、审计、物价、体育、广播电视、房管、外事侨务、民族宗教、对台、人防、档案、机关事务、民兵预备役、残联等部门工作取得了新的成绩。

“非典”防控成效显著。面对突如其来的“非典”疫情，我们坚决贯彻中央和省关于抗非工作的一系列重大决策部署，坚持一手抓“非典”防治，一手抓经济建设，构筑了群防群控的防治网络。各级领导干部深入一线，身先士卒，靠前指挥，充分发挥了表率作用。广大基层组织和共产党员冲锋在前，迎难而上，充分发挥了战斗堡垒和先锋模范作用。广大医护人员恪尽职守、无私奉献，充分展示了救死扶伤、不怕牺牲的精神风貌和道德情操。全市人民群众发动广泛，人人参战，构筑起抗击“非典”的坚强屏障，取得了“非典”防治的阶段性重大胜利，全市没有发生一例“非典”病例。“非典”过后，我们随即把防治工作从应急状态转入常态管理，制定“非典”应急处理预案，加强公共卫生体系建设，建立公共卫生及其他各种应急机制的长效机制，增强了应对突发事件的能力。

精神文明和民主法制建设不断加强。深入贯彻公民道德建设实施纲要，群众性精神文明创建活动取得显著成效。认真执行市人大及其常委会的各项决议、决定，自觉接受人大依法监督、政协民主监督，办理人大代表建议303件，政协委员提案300件，办结率100%。提请市人大常委会审议地方性法规5件、制定规章15件。深入开展普法教育，市民法制观念不断增强，村民自治建设和社区建设有了较大进展。全面开展效能建设，认真落实行政执法责任制和执法违法责任追究制，依法行政水平不断提高。积极开展“双拥”活动，军政、军民关系进一步密切，第四次荣获“全国双拥模范城”称号。加强社会治安综合治理，深入开展“严打整治”专项斗争，社会治安状况进一步好转。不断强化安全生产的各项措施，安全生产形势基本稳定。认真落实信访工作责任制，妥善化解社会矛盾。积极推进政务公开，深入开展反腐败斗争，反腐倡廉取得明显成效。

（六）就业和社会保障工作稳步推进，城乡人民生活不断改善

切实帮助解决困难群众的生产生活问题，以扶贫帮困、农村基础设施建设、完善城市功能、改善生产生活环境为主要内容的10件实事件件落到了实处。认真抓好促进就业、再就业工作，全市发放下岗失业人员《再就业优惠证》5.2万个，下岗失业人员减免税486.38万元，减免收费116.1万元，发放下岗失业人员小额担保贷款1200万元，加大了失业保险基金的征缴力度，发放失业保险金2100万元。新增城镇就业岗位3.65万个，解决城镇失业人员就业2.24万人，下岗失业人员再就业1.82万人(其中“4050”人员5600人)，农村富余劳动力转移1.03万人。加强了市区劳动力市场和社区劳动保障所的建设，在71个街道办事处和乡镇建立了劳动保障所，将427个社区低保工作人员整合纳入劳动保障所管理。完善了低保措施和工作体系，全市共发放低保金4700万元，享受低保人数达7.89万人，做到应保尽保。城镇登记失业率控制在3.9%以内。落实了中央调整机关事业单位职工工资标准和增加离退休费的政策。加大了对18个重点贫困乡449个贫困村的扶贫开发力度。城镇居民人均可支配收入和农民人均纯收入都有较大幅度增长，人民生活水平有了新的提高。

过去的一年，我们走过了不平凡的历程，面对众多不利因素，我们战胜了各种艰难险阻，各项工作取得了新的进展，成绩来之不易。这是省委、省政府、市委正确领导和市人大、市政协有效监督的结果；是各级各部门解放思想，与时俱进，开拓创新，提高效能的结果；是全市人民团结一致，顽强拼搏，真抓实干的结果。在此，我代表市人民政府向全市广大干部群众致以崇高的敬意!向驻筑解放军、武警部队、公安干警，向各民主党派、工商联、无党派人士和人民团体，向所有关心支持贵阳建设和发展的各界朋友，表示衷心的感谢!

在充分肯定成绩的同时，我们也清醒地看到，在经济社会发展中还存在一些困难和问题。主要表现在：经济总量比较小，经济结构不尽合理，经济增长的基础还不够稳固，城乡二元结构明显；农村部分地区扶贫攻坚难度大，农民增收困难；就业和再就业形势严峻，影响社会稳定的因素还不少；经济发展的环境仍不够宽松，一些深层次矛盾尚未解决，改革开放的任务艰巨。对此，我们将在今后的工作中高度重视，采取切实有效措施，认真加以解决。

二、2004年主要工作任务

今年是实施“十五”计划的关键年，也是全面落实十六大和十六届三中全会精神，实施大开放、建设“大贵阳”的重要一年。根据中央和省经济工作会议的精神，今年全市工作的总体要求是：以邓小平理论和“三个代表”重要思想为指导，全面贯彻十六大、十六届三中全会、省委九届四次全会、中央经济工作会议、省经济工作会议精神，坚持以人为本，树立全面、协调、可持续的发展观，按照稳定政策、适度调整，深化改革、扩大开放，把握全局、解决矛盾，统筹兼顾、协调发展的思路，以“抢抓机遇、加快发展”为主题，围绕建设“大贵阳”的目标，继续实施“环境立市、工业强市、科教兴市”战略，大力推进城镇化、新型工业化、农业产业化进程，实施好七大工程，保护好、引导好、发挥好各方面加快发展的积极性，切实把工作重点转移到转变增长方式、提高增长质量和效益上来。按照持续快速协调健康发展的要求，确保经济社会发展再上新台阶。

今年经济和社会发展的预期目标是：全市生产总值增长12%，其中：第一产业增加值增长5%，第二产业增加值增长13.5%(工业增加值增长13%)，第三产业增加值增长11.5%；财政总收入增长12%，地方财政收入增长12%；固定资产投资增长20%；社会消费品零售总额增长12%；进出口总额增长15%；实际利用境内外资金增长15%，其中实际利用外资增长12%；城镇登记失业率控制在4.5%以内；城镇居民人均可支配收入增长8.5%；农民人均纯收入增长6.5%；人口自然增长率控制在8‰以内。

市委、市政府为民办好的十件实事是：

1、增加城镇就业岗位3.8万个；建立、完善农村特困群众救助制度；启动贵阳市工伤保险制度。

2、实施农村现代远程教育工程，在100个边远贫困村修建远程教育接收点，建成884个村级小学卫星接收站，完成86个乡镇中学多媒体教室的建设；完成250个自然村通广播电视工程；完成200个村地面卫星接收站“模改数”工程。

3、建成贵阳人才大市场；实施科技强市“百千万”培训工程(百名非公企业高级科技人才培训，千名制造业信息化科技人才培训，万名贫困农民科技培训)。

4、建成贵阳城市公共安全突发事件网上应急处理系统；完成7个区、市、县疾控中心建设。

5、改造16条城市道路、5条城市大沟；开工建设经济适用住房100万平方米，竣工60万平方米。

6、新增城市绿地30万平方米；完成“二环”林带建设9万亩；拆除违法建筑25万平方米；启用5个停车场；对城区3750座化粪池和排污管道进行全面改造，建成贵阳市医疗垃圾焚烧场。

7、改造农村茅草房12600户，全面消灭农村茅草房；解决农村6.5万人饮水困难；完成500公里县乡公路改造、1000公里贫困村公路改造和13个铁路道口平改立工程；在农村新建沼气池20000口，改圈20000个，改厕20000个，改灶20000个，改厨20000个。改造农户进户路20000户；建设80个村级宣传文化室。

8、启动金阳新区第二个大型住宅楼盘；基本建成金阳观山公园、金阳石林公园；完成金阳医院主体工程和金阳商贸城主体工程。

9、启动贵阳一中搬迁，完成贵阳三中搬迁；在金阳新区碧海花园建成小学、中学、幼儿园各一所；完成市民健身中心主体工程，建成科技大厦；开放贵阳群众艺术馆、美术馆。

10、建成70个社区居委会办公用房；解决困难群众廉租住房600户；完成20个乡镇司法所建设。

为实现上述目标任务，我们将重点抓好以下工作。

（一）进一步加大投资力度，努力扩大消费需求，拉动经济快速增长

紧紧抓住国家实施积极财政政策和中央国债资金向西部、三农倾斜的机遇，千方百计谋划项目，实施“项目带动工程”，努力扩大投资，确保固定资产投资比去年净增50亿元以上。

进一步加大旧城改造力度。继续加大城市中心区道路改造的力度，完善城市道路交通网络，改善区域交通，打通主次干道，缓解片区交通，确保完成珠江路、友谊路、观水路、贵乌路等16条城市道路改造，加快贵开公路的建设。继续做好中日环境合作示范6个项目的建设，全面完成南明河环境综合整治系列工程，完成5条城市排污大沟改造，逐步完善我市城市排水和污水处理系统，争取利用日本政府贷款1亿美元治理贵阳水污染项目获得国家的批准。加快推进循环经济型生态城市的建设步伐，提高城市品位，争创联合国人居环境奖。

强力推进金阳新区建设。运用市场新机制引进外资、吸收民资，聚合新型产业、聚集人气，力争实现新区建设“一年一个样，三年大变样”。进一步加快金阳新区40平方公里基础设施建设和公共公用设施建设，开工建设一批标志性建筑工程，推进金阳商贸走廊、体育中心、学校、医院等项目建设，完成金阳市级行政中心二期工程建设，推进新区住宅产业化进程，加快土地储备和挂牌出让进度，加大房地产开发力度。运用市场化运作的方法，加大招商引资力度，吸引国内外资金参与新区建设，力争完成固定资产投资80亿元。

不断加快小城镇建设步伐。以县城和条件较好的城镇为重点，多渠道加大基础设施建设投入，努力扩大县城规模。切实搞好小城镇综合体制改革，坚持特色立镇、产业立镇，着力发展一批贸易型、工业型、旅游型特色小城镇，并以中心小城镇为载体，大力发展二、三产业，增强小城镇的经济支撑能力，实现建一个小城镇，活一片经济，兴一方产业，富一方百姓。

千方百计提高人民群众的消费能力。下大力帮助城乡困难群众解决基本生活问题。继续通过减轻农民负担，加快推进农业结构调整和农业产业化经营，促进农村富余劳动力转移等措施，努力增加农民现金收入，拓宽农村消费市场，搞好城乡商品销售网络的衔接，提高农村消费能力。进一步加快市场体系建设步伐，切实搞好市场规划和引导，不断提升现有商品市场和商业网点的规模、档次、品位和经营水平，积极推进与国内外著名商业企业的合作。大力发展普通商品房和经济适用房，抓紧做好居民房屋产权证发放工作，全面放开住房二级市场，鼓励居民按揭贷款购房，进一步促进房地产业和相关产业的发展。结合纠正部门和行业不正之风，进一步清理在汽车、建设、教育、医疗等领域的不合理收费。积极引导和促进居民扩大住房、家用轿车、电信、旅游、文化、教育、体育、休闲娱乐和其他服务性消费。大力改善消费环境，逐步扩大消费需求。进一步清除限制消费的各种障碍，完善扩大消费的政策措施，培育新的消费热点。

（二）切实解决好“三农”问题，千方百计增加农民收入，统筹城乡区域的协调发展

切实推进农业结构调整。进一步加大对农业产业化龙头企业的扶持力度，重点扶持无污染、覆盖面广、带动力大的产业化重点项目，延长产业链，提高农产品的附加值，促进农业增产、农民增收。继续调整种植结构，在保护和提高粮食综合生产能力的基础上，突出发展高效经济作物和特色蔬菜等产业，依靠科技提高农业比较收益，使经济作物生产比重进一步加大，力争粮食总产量达到55万吨，商品蔬菜上市量34万吨，烤烟产量30万担。加快畜牧业的发展，不断提高畜牧业产值占农业总产值的比重。大力发展都市农业，重点抓好观光农业、蔬菜基地、中药材基地的建设，以基地带动农户，积极推进农产品质量标准体系建设，启动农产品市场准入制。健全农业科技支撑服务体系，争创国家级农业科技园区，提高农业科技含量。完善农村生产、销售服务体系，建立健全各类行业协会中介组织，提高农产品竞争力。积极发展农村专业协会，推进农业专业化生产、市场化经营。深化改革，大力发展混合经济和股份制经济，推进乡镇企业二次创业和改造升级，重点鼓励乡镇企业发展农产品加工、储藏、保鲜、运输业和农村服务业，提高对农民的带动和吸纳能力。确保实现乡镇企业增加值增长15%，力争增长17%的目标。

加强农业农村基础设施建设，切实改善农村生产生活条件。

围绕农村水、电、路、气、房，大力实施“四改一气一种一养”(改圈、改厕、改厨、改灶、沼气池、种植业、养殖业)工程，完成中低产田土改造5万亩，治理水土流失面积25平方公里，新建11座小型水库，解决6.5万农村人口饮水困难。启动10个生态村试点建设，完成500公里县乡道路改造，建成1000公里通村公路，完成茅草房改造12600户，全面消灭茅草房。抓好农村电网改造，实现城乡电网同网同价。继续实施好村村通广播电视、通电话工程。

加大扶贫开发力度，促进农村劳动力转移。建立、完善农村特困群众救助制度，落实对鳏寡孤独、因病因残丧失劳动能力的农村特困人员进行定期救助的政策。通过产业培育、退耕还林还草还药、劳务输出、社会帮扶等措施来消除贫困、稳定致富。着力实施农村党员干部现代远程教育工程，加强农村劳动技能培训和素质培训，完成非农技能培训3.5万人，引导农村富余劳动力合理有序转移，今年新增有序转移1.4万人。加强小城镇规划建设，完善功能，结合移民迁建，引导农民向小城镇集中，吸纳农产品加工业和涉农服务业向小城镇聚积。继续清理各种乱收费，切实解决拖欠克扣农民工工资、劳动安全和职业病防护无保障等突出问题，建立欠薪保障制度，在户籍、子女就学等方面给予农民工平等待遇，保护好农民工的合法权益。

进一步深化农村各项改革，促进农村经济稳步发展。长期稳定并不断完善土地承包经营制度，稳健推进土地经营权流转，科学规划、合理利用土地资源。继续落实好农村税费改革试点的各项政策，加快形成以农业税及附加和严格规范的“一事一议”筹资筹劳为主的农业税费制度框架，切实减轻农民负担。规范村级财务管理，加强涉农收费管理，继续落实和完善涉农价格和收费公示制。

大力发展县域经济，促进区域经济统筹协调发展。充分发挥区县的区位、市场和资源的比较优势，用改革的思路、开放的办法，完善加快区县经济发展的政策措施，支持区县经济的发展。按照省的统一部署，启动实施第二轮经济强县建设，进一步巩固云岩、南明、白云、乌当四区目前的优势；加大花溪、清镇、开阳建设经济强县的力度，力争排位前移；力争小河、修文、息烽进入第二轮“建强”行列。支持有条件的区、市、县保持高于全国、全省、全市的发展速度。合理放开经济管理权限，让出更大的发展空间，适当下放事权，增强统筹能力，增强县域经济发展自主权。

（三）实施“结构调整工程”，着力提高工业经济运行质量和效益，大力发展服务业着力提高工业经济运行质量和效益

进一步加快高附加值工业的发展速度。把国有企业的技术改造放在突出位置，积极采用高新技术和先进适用技术，优化提升冶金、化工、卷烟、能源等现实优势产业，大力发展现代中药、食品工业、电子信息、新材料等具有竞争优势的行业，提高装备水平和产品质量。大力发展高新技术产业，不断提高高新技术产业增加值在整个工业增加值中的比重。全力支持高新技术开发区金阳知识产业园区的建设，引进和开发一批电子信息、新材料、光机电一体化等高新技术产业和产品，加快发展南方汇通微硬盘、反浸透膜及环保设备、振华电子原器件、海信工业园二期、华城公司楼宇自控设备等高新技术项目，扶持企业加快GMP改造步伐，争取6月30日按国家要求完成改造。重点抓好博士公司电脑耗材、特种陶瓷等新材料，广航有色金属铸造及精密冷挤压中心等铝深加工，詹阳公司高速挖掘机等装备国产化项目。大力支持中铝公司贵州铝厂第四期17万吨电解铝项目和年产120万吨氧化铝项目，磷铵、三聚磷酸钠项目及险峰厂高精度数控机床技改、D28柴油机、汽车零配件等新项目建设进度。加快企业信息化带动工业化的建设步伐。大力发展食品工业。全力抓好“1050”工程，在抓好已实施项目竣工验收工作的基础上，加大新开工项目启动力度，为“十五”期间完成“1050”工程的目标打下坚实基础。积极做好协调服务工作。着重抓好重点物资铁路运输和重点企业电力供应的协调。拓宽融资渠道，强化企业信用，积极协调金融部门对工业项目的信贷支持，运用市场机制加大筹资力度，带动银行、企业和社会各方资金投入。进一步加强对企业的跟踪服务，努力提高企业运行的质量和效益。

大力发展服务业对扩大消费面、增加消费项目、促进经济增长有着重要的推动作用。大力发展现代物流业，抓紧编制《贵阳市现代物流发展规划》，借助电子信息技术和先进的经营管理方式，加快对传统流通业的改造步伐，发展连锁经营、物流配送、物流园区。调整优化市场布局，逐步完善以大十字为中心的中央商务区功能，抓好12个重点市场的建设和改造工作，重点发展专业特色鲜明、集散功能较强、连接城乡、辐射全省及西部的大型现代批发市场。加快国有大型商业企业改革、改制步伐，进一步盘活存量，推动国有资产有序转移。积极培育新兴服务业。发展家政服务、社区服务、中介服务、非义务教育等新兴服务业，拓宽服务业的发展空间。大力发展旅游业。整合全市范围内的旅游资源，规划旅游景区建设，提高旅游的文化品位，努力打造并形成一批有特点、有吸引力的精品旅游线路。加大旅游开发建设投入，逐步完善旅游基础设施，真正形成几个在国内外有较大影响力的旅游品牌。强化旅游行业管理，继续开展旅游市场治理整顿工作，不断提高服务质量，确保实现旅游总收入增长15%以上，接待国内外旅游者增长15%以上。加快信息化建设步伐。结合市级行政中心搬迁，在更高起点上加快电子政务和贵阳市地理信息系统及工商、税务、公安信息化建设，实施“金税”、“金盾”工程。进一步完善就业、社保、便民服务等信息网络建设，建成贵阳城市公共安全突发事件网上应急处理系统。

（四）进一步加大改革力度，大力发展非公有制经济，努力扩大对内对外开放

大力推进国有企业改革。以产权制度改革为突破口，力争用三年左右时间实现国有资产从一般竞争性领域退出。今年从全市国有企业中选择30%—40%条件成熟的企业作为第一批实行资产重组和转让的重点企业，下大力引进实力较强的知名企业、上市公司对这些企业实施资产重组，推进我市国有企业结构的调整。充分利用国家兼并破产的有关政策，对已列入贵阳市企业破产计划的11户国有大中型企业，积极争取国家和省的支持，今年做好3户企业的破产申报工作，力争列入国家政策性破产计划。对已关停或半关停的中小企业，进一步加强政策指导，引导企业转变观念，鼓励非公有制经济介入，在妥善处理好资产处置、人员安置等问题的基础上，采取重组、联合、兼并、股份合作制、出售等多种形式，全面放开搞活国有企业。积极推进国有企业股份制改革。对部分规模较大、资产优良、主业突出的国有企业，除国家有明确规定的外，逐步改制成为由国有资本、集体资本和非公有资本参股或控股的股份制企业；对已进行股份制改造的国有企业进行评估检查，对国有股权比重过大的企业，根据具体情况采取吸纳其他非公有资本入股或转让部分股权的方式，改善股权结构。进一步深化国有企业经营机制改革。按照建立现代企业制度的要求，进一步完善企业公司法人治理结构、财务制度和决策机制。继续深化配套改革。进一步充实和完善国有企业离退休人员托管中心，加快社会保障体系建设，减轻国有企业冗员负担。通过资本市场筹集资金，调整企业资产负债结构，减轻国有企业债务负担，为国有企业的改革和发展创造良好的外部环境。

健全和完善国有资产管理体制。组建市国有资产管理委员会。按照十六届三中全会精神和国务院《国有企业监督管理暂行办法》的要求，抓紧完善权利、义务和责任相统一，管资产与管人、管事相结合的国有资产管理体制和运行机制；探索国有资产监管和经营的有效形式，积极稳妥地对国有资产经营公司和部分大型企业实行授权经营；建立健全经营者激励机制，推行企业经营者年薪制，进一步落实经营业绩考核、国有资本收益收缴管理办法和国有资本经营预算制度，强化和规范管理者的责任，督促企业实现国有资产保值增值。

进一步深化投融资体制改革。落实企业的投资主体地位，逐步形成投资主体自主决策、银行独立审贷、融资方式多样、中介服务规范、市场有效配置、宏观调控有效的新型投资体制。合理划分投资管理权限，规范政府部门行为，改革投资项目审批制度，减少审批环节，下放审批权限。加快形成投资主体多元化、筹资方式多样化、政府调控间接化的运行机制，引导投资需求合理增长，扩大社会投资特别是民间投资领域；通过规划和政策指导、信息发布以及规范市场准入，引导社会投资方向，抑制无序竞争和盲目重复建设。

不断深化财税金融体制改革。加强税收征管，理顺税收征管体制，坚持“依法治税，应收尽收，坚决不收过头税”的原则，禁止越权减免税和各种形式的买税卖税行为，积极做好税制改革的各项准备工作。进一步加快实施以部门综合预算、国库集中收付、政府采购为核心的财政改革，不断强化支出管理，提高财政资金的使用效益。按照国家的规范化标准和要求，进一步细化预算内容，完善部门预算管理办法。积极推进国库集中收付制度改革试点，逐步规范收入收缴和支出拨付程序，进一步加大国库集中支付力度。压缩一般性支出，重点保证工资支付、社会保障、贫困救助、正常运转和“三农”、公共卫生体系建设及重点建设项目的资金需要。认真贯彻执行《政府采购法》，不断完善政府采购制度，规范政府采购行为，增强政府采购的透明度，提高政府采购的效率。深化“收支两条线”管理改革，认真清理收费项目和标准，规范行政事业性收费、罚没收入和预算外资金管理。大力发展区域金融，做大做强贵阳市商业银行，争创金融安全区，进一步发挥金融促进发展的作用。按照省的统一安排，积极推进农村信用社的改革工作，争取在部分区县组建农村合作银行。

进一步完善和规范市场体系。抓紧实施统一的劳动力市场建设规划，建成贵阳人才大市场，整合城乡劳动力市场和人才市场。加快产权交易市场建设，推动国有产权进场交易；规范发展资本、技术等要素市场。深化价格改革，建立价格和收费公示制度。制定“信用贵阳”建设总体方案和实施办法，加快建立信用征集体系，建立和完善失信惩戒机制。以直接关系人民群众身体健康和生命安全的食品、药品为重点，深入开展整顿和规范市场经济秩序工作，依法严厉打击制假售假和商业欺诈行为。

大力发展非公有制经济。鼓励非公有制企业承包、租赁、收购、兼并国有、集体或其他企业，更多地进入高新技术产业、新型服务业和社会公益事业等领域。继续贯彻执行市委〔2003〕1号文件精神，认真落实发展非公有制经济的各项优惠政策，加大对非公有制经济的扶持力度。完善中小企业信用担保体系，协调好企业与银行的关系，加强对非公有制经济的信贷投入，协调解决好非公有制经济在市场拓展、信息咨询、人才培训和社会保障等方面的实际困难。充分发挥工商联、侨联、台办的职能作用，加强对港澳台商、海外投资者、非公有制企业的联系和服务。支持和引导各类商会、行业协会及其它中介组织为非公有制企业搞好服务。规范行政执法，严肃查处损害非公有制企业合法权益的行为。引导非公有制企业提高素质，依法经营，照章纳税，保障职工合法权益。

大力加强区域协作。着眼于拓展国际国内两个市场，利用境内境外两种资源，引进国内国外两类资金，争取在更大、更广领域和更高层次上参与国际经济分工与经济技术合作。继续加强与大连、青岛、宁波、深圳四城市的联合，扩大与南宁、昆明两市的经济交流与合作，促进三地优势互补，共同打造南贵昆经济区，推动区域协作向深层次、多渠道、全方位统筹协调发展。

努力扩大对外开放。不断强化招商引资工作，进一步完善招商引资的优惠政策体系，建立健全招商引资责任制和激励机制，实施“环境创新工程”，在全市形成重商、亲商、安商的浓厚氛围，打造投资成本最低、回报最快、办事效率最高、信誉最好的招商引资环境，做到以情招商、以诚招商。创新招商方式，实行会议招商和上门招商相结合，广告招商和网上招商相结合，直接招商与代理招商相结合。进一步明确招商引资的重点产业、区域和目标企业，把金阳新区开发建设和国有企业改制重组、企业国有产权、股权转让项目作为吸引外资的重点，广泛吸引国内外知名客商，务求取得大的突破。抓住我国沿海地区资本向西部梯度转移的良好机遇，充分利用我市磷、铝、天然中药材、能源和旅游资源丰富的优势，重点面向环渤海、长江三角洲、珠江三角洲经济圈等国内发达地区招商，积极参与泛珠江三角洲经济区建设。切实做好招商项目的跟踪落实，将招商协议变为正式项目，确保招商落到实处，引资见到成效。

积极开展对外贸易。积极应对国家出口退税政策变化的新形势，落实和完善促进进出口的各项政策，充分调动企业扩大出口的积极性。今年我们将力争在全市外贸出口数量和质量上有一个新的突破。努力巩固原有出口市场，开拓新市场，积极支持骨干企业扩大出口，重点支持绿色食品、机电、化工等产品的出口。

加强对重点出口企业的联系和重点出口产品的跟踪服务，改善出口商品结构和环境，培育新的出口增长点。努力为更多的企业争取进出口自营权。今年力争市属企业外贸进出口额增长15%以上。

（五）以创建全国文明城市为目标，塑造现代化城市形象，提升城市管理水平

按照建设“大贵阳”的目标定位，不断提高城市绿化、净化、美化、亮化水平，改善人居环境，提升城市品位。进一步加强南明河综合治理，推动城市管理上台阶。以实施南明河三年变清系列工程为契机，按照《南明河保护管理办法》，依法加强对南明河沿岸的管理，以此带动整个城市管理水平不断提高。切实保护城市公用设施，逐步完善长效管理机制。积极探索和完善城市管理考核指标体系，进一步明确市、区、街道(社区)在城市管理和保护城市公用设施中的责任，强化基层基础工作，严格考核，使长效管理措施真正落到实处。在开展相对集中行政处罚权试点工作的基础上，全面推进城市综合执法，努力营造优良的执法环境。坚决拆除各类违章建筑，完成3750座化粪池改造工作。

大力加强交通秩序、市容环境卫生整治，不断提高市民文明素质和城市文明程度。继续下大力搞好市容环境和交通秩序综合整治。重点抓好背街小巷、城郊结合部脏、乱、差的治理。结合实施畅通工程，针对我市行车秩序、停车秩序、行人秩序和占道经营等方面存在的突出问题，加大整治力度，完善交通设施，提高城市交通管理水平。

加快市政公用事业的改革步伐。进一步健全土地收购储备制度，完善土地市场交易规则，做好经营性土地招标拍卖和挂牌出让工作，充分挖掘城市资源，积极运用市场机制的办法运作城市建设和城市管理项目。继续推进市政基础设施由单纯的政府投入向多元化投入转变，市政公用设施由无偿使用向有偿使用转变，公用事业由福利服务向经营服务转变，逐步实现市政公用事业投资多元化、运营市场化、服务社会化。积极推进公交公司、自来水公司及新建污水处理厂等市政企业的改革。

加强生态环境保护，促进城市可持续发展。以创建国家园林城市为目标，加快中心区绿地建设，新增城市绿地30万平方米，建设二环城林带9万亩。合理开发和保护水、耕地、矿产、森林等资源，提高资源利用效率，实施节水措施。加大对无证开采、乱采滥挖、采富弃贫、破坏资源等行为的整治力度。加强酸控区和重点流域的综合整治，开展饮用水源地周边污染源的集中治理，着手建设危险废物处置中心和医疗废弃物处置中心。做好固体废物综合利用、工业企业污染综合整治工作，促进城市生态环境的良性循环。

（六）实施“民心工程”，切实解决关系人民群众切身利益的问题，不断提高人民生活水平

把促进就业放在突出位置，努力扩大就业，不断增加城乡人民的收入，提高全市人民的生活水平和质量。牢固树立“以发展促就业，以就业促发展”的理念，把大力发展服务业、非公有制经济和推动小城镇建设作为扩大就业的主渠道来抓。加大再就业补助资金的投入力度，全面落实促进就业的各项优惠政策，把财政资金、信贷支持和税费减免等政策真正落到实处。以培育和发展劳动力市场为重点，大力开发就业岗位，不断改善就业环境，进一步做好有劳动能力和就业愿望的下岗失业人员的就业服务工作。积极引导和组织下岗失业人员转变择业观念，强化就业培训，提高择业技能，开展就业援助，推行灵活就业，实现经济协调发展和劳动者充分就业的双重目标，力争全年新增就业岗位3.8万个，下岗失业人员再就业1.65万人。

进一步推进社会保障体系的建设。继续巩固“两个确保”，加强“三条保障线”的衔接。切实解决好国有企业下岗职工出中心和国有企业破产重组职工安置的经济补偿及社会保险关系接续等问题。不断完善城镇职工基本养老保险、失业保险制度，继续扩大社会保险覆盖面，提升社会保险统筹层次，努力实现市区社会保险管理体制的整合。坚持“三改并举”，着力推进医疗保险制度改革，启动职工工伤保险，推进“金保”二期工程建设，建成延伸区、市、县、街道、镇和社区劳动保障服务网络并投入试运行。探索建立农村养老和最低生活保障制度，进一步健全和完善城乡居民最低生活保障制度，规范管理，提高水平，做到应保尽保。

妥善解决好困难群众的实际问题。把解决好困难群众的生产生活问题，作为实践“三个代表”重要思想要求最实际、最紧迫的行动，作为“民心工程”、“凝聚力工程”，时刻把人民群众的安危冷暖放在心上，真心实意地帮助困难群众排忧解难，实实在在地为群众谋利益。建立完善灾害救助、社会救济、社会互助工作体系和经常性工作机制，切实做好扶贫开发、救灾救济、拥军优属等各项工作。规范企业工资支付行为，在效益增长的基础上，提高企业工资水平，严禁企业违法拖欠和克扣工人工资。保证农村中小学教师工资按时足额发放。高度重视和关注弱势群体，切实解决城乡困难群众的生产生活问题，在就医、子女入学、住房、法律援助等方面给予优惠和照顾。广泛开展扶贫济困送温暖活动，让群众切身体会到党和政府的关心与温暖。

（七）加强政治文明和精神文明建设，实施“先进文化工程”，统筹经济社会的协调发展

全面加强精神文明建设。深入开展邓小平理论、“三个代表”重要思想和党的十六大精神宣传教育，树立中国特色社会主义共同理想。认真贯彻公民道德建设实施纲要，加强社会公德、职业道德、家庭美德教育，倡导诚实守信。深入开展创建文明城市活动，巩固和发展创建成果。坚持先进文化前进方向，大力发展文化事业和文化产业，繁荣和发展哲学科学。深化文化体制改革，繁荣文艺创作，努力创作出一批文艺精品力作。广泛开展群众文化活动，加大对公益性文化事业的投入，搞好广播电视网络的建设，实施好“村村通广播电视”工作。加强文化市场管理，大力发展文化产业。抓好科教、文体、法律、卫生入乡村进社区活动，不断提高公民的思想道德和科学文化素质。广泛开展双拥共建活动，争创全国双拥模范城。

积极推进科技进步和技术创新。继续深化科技体制改革，大力实施以企业为主体的技术创新工程，增强企业创新能力。认真落实科技成果转化为生产力的各项政策，促进科技成果的转化，提高科技对经济增长的贡献率。建立和完善与全市经济社会发展相适应的科技创新体系，建立健全各类孵化点，充分发挥生产力促进中心的作用，进一步整合科技资源，优化资源配置，做好重点科技成果推广工作。强力推进高新技术产业化。进一步加大科普工作力度，建好科普宣传栏。

大力发展教育事业。进一步巩固和扩大“两基”成果，全面加强素质教育，发展高中教育和鼓励发展民办教育，完成高中扩招任务，力争高中阶段毛入学率达到60%以上。逐步解决市区热点学校班容量过大和薄弱学校门庭冷落问题。扎实做好金筑大学、贵阳师专合并申办“贵阳学院”的各项工作。积极推进教育信息化进程，建成贵阳教育城域网。加快教育基础设施建设，使“退高进初”迈出实质性步伐。贵阳一中、贵阳三中新址实现搬迁，开工建设贵阳二中、六中、九中示范性高中新校和金阳新区大学城。

大力实施“人才工程”。牢固树立人才是第一资源的观念，加大人才培养、使用和引进力度，推进人才整体开发，实施科技强市“百千万”培训工程，建设贵阳人才大市场。强化用人单位在人才培训中的主体地位，鼓励在职学习。努力用好现有人才，积极引进紧缺急需人才，健全完善人才引进政策，创新人才引进机制，改革人事制度和分配制度，营造吸引人才、留住人才、用好人才、爱护人才的良好环境，集聚更多人才为贵阳发展服务。

积极推进卫生、体育等社会事业发展。大力发展公共卫生事业，进一步深化医药卫生体制改革，认真落实预防和控制“非典”等各类传染病的工作预案，加快建立传染病防治的长效机制，完成7个区、市、县疾控中心建设。健全市、县、乡三级疾病预防控制体系、医疗救治体系和卫生监管体系，完善城市、农村卫生服务体系和妇幼保健体系，积极稳妥地扩大新型合作医疗试点工作，逐步建立农村医疗保障体系。广泛开展全民健身运动，提高竞技体育水平。加大对体育设施的投入力度，建成全民健身中心主体工程，搞好金阳体育中心的规划、设计等前期准备工作，今年开工建设。进一步加大人口与计划生育工作力度，强化人口与计划生育工作责任制，稳定低生育水平，不断提高出生人口素质，人口自然增长率控制在8‰以内。要进一步做好老龄工作，提高老龄人口的社会化服务水平。继续做好统计、审计、民族宗教、外事侨务、人防、档案、地方志等工作，充分发挥工、青、妇等群众团体、民兵预备役、武警消防部队的作用，调动社会各界参与现代化建设的积极性。

大力实施“平安贵阳工程”，进一步强化社会治安综合治理。坚持打防结合，预防为主，建立和完善社会治安防控体系。

深入开展“严打”整治斗争，严厉打击各种刑事犯罪和经济犯罪，坚决扫除“黄、赌、毒”等社会丑恶现象，依法处理和取缔“法轮功”等邪教组织。积极预防和妥善处理群体性事件，加强信访接待和疏导教育工作，把不稳定因素化解在萌芽状态，切实维护社会稳定。

强化安全生产责任制，强化交通、消防、煤矿、危险化学品等的安全生产工作。全面落实煤矿业主和管理者管理责任，严防非法无证煤矿死灰复燃，妥善解决农民生产生活用煤问题。加大对安全事故的查处力度，严格执行安全生产和安全事故责任追究制，及时消除事故隐患，有效遏制重特大事故，力争安全事故起数和死亡人数比去年有所下降，确保人民群众生命和财产安全。

（八）切实加强政府自身建设，努力提高工作水平和效能

面对新的形势和任务，我们必须切实加强政府自身建设，必须树立正确的政绩观和科学的发展观，必须做到求真务实，改革不适应形势发展的行政方式，尽快形成行为规范、运转协调、公正透明、廉洁高效的行政管理体制和运行机制，树立为民、务实、清廉的形象。

深化行政体制改革，建设高效服务型政府。进一步深入学习领会党的十六届三中全会和中央、省经济工作会议精神，树立强烈的机遇意识、改革开放意识、创新意识和服务意识，增强建设“大贵阳”，实施跨越式发展的使命感、责任感和紧迫感，不断研究新情况，总结新经验，解决新问题。不断加强学习，提高自身素质，深化对市情的认识，正确把握贵阳经济社会发展的基本规律，不断提高“五个统筹”、“五个坚持”的能力，提高驾驭市场经济和应对复杂局面的能力，以创新的思路和举措，全力抓好各项工作，切实把政府职能转变到经济调节、市场监管、社会管理和公共服务上来。进一步加大行政审批制度改革力度，除国家规定的关系经济安全、影响环境资源、涉及整体布局的重大项目和政府投资项目及限制类项目外，其他项目的审批制改为备案制。抓好《行政许可法》的培训和学习，抓紧清理、修改和废止与《行政许可法》不相适应的行政规章和规范性文件，加快配套制度建设，保证今年7月1日《行政许可法》的顺利实施。对关系人民群众切身利益的重大决策事项实行听证制度，增强行政透明度，提高民主决策、科学决策水平。

全面推进民主法制建设。坚持向市人大常委会报告工作、向市政协通报情况制度，自觉接受市人大的法律监督和市政协的民主监督。高度重视人大代表建议和政协委员提案的办理工作，把建议和提案作为政府知晓民情、了解民意的重要渠道。加强与工会、共青团、妇联等人民团体和民主党派、工商联、无党派爱国人士的联系。完善重大决策规则和程序，建立健全深入了解民情、充分反映民意、广泛集中民智、切实珍惜民力的决策机制，推进决策的民主化、科学化。深入开展“四五”普法教育，增强全市人民的法律意识和法制观念。适应我国加入世贸组织和完善市场经济体制的需要，进一步做好地方行政规章和规范性文件的废、改、立工作。加强基层政权建设，完善村民自治、居民自治和职工代表大会制度，推行政务公开、村务公开、厂务公开、校务公开等基层民主管理制度，扩大基层民主。

继续加强行政效能建设，切实转变政府职能。努力营造廉洁高效的政务环境、规范有序的市场环境和富民安民的生活环境。积极创建学习型机关，全面提升公务员队伍的整体素质。切实改进领导方式和工作作风，大力精简会议和文件，经常深入基层开展调查研究，体察民情，吸纳民意，增强执政为民的公仆意识。强化行政执法监督和机关效能建设，规范执法行为。推行行政过错责任追究制度，提高行政管理质量。加快政务信息化建设，增强公务员的信息技术应用能力，提高行政效率，监管能力和服务水平。

坚持艰苦奋斗，做到廉洁行政。坚持不懈地开展党风廉政建设和反腐败教育，增强清正廉洁、拒腐防变意识。坚持标本兼治，加大从源头上预防和治理腐败的力度，大力纠正部门和行业的不正之风。针对人民群众反映强烈的突出问题，深入开展专项整治，坚决查处贪污腐败、以权谋私等违法违纪案件，切实以清新廉洁的政风，带动整个社会风气的根本好转。

各位代表，建设“大贵阳”的目标催人奋进，加快发展、实现“两个率先”的历史任务十分艰巨。让我们团结在以胡锦涛同志为总书记的党中央周围，以党的十六大和十六届三中全会精神为指导，高举邓小平理论伟大旗帜，全面实践“三个代表”重要思想，在省委、省政府和市委的正确领导下，与时俱进，开拓创新，励精图治，扎实苦干，为全面完成今年的各项目标任务，加快贵阳经济社会跨越式发展而努力奋斗!
